# Crystallization of Vanillin Isomers in Carboxymethyl Chitosan Gels

**DOI:** 10.3390/gels11040285

**Published:** 2025-04-11

**Authors:** Lin Zhang, Xiaoling Lu, Hao Li, Hongjie Chen, Qi Yin, Xuehan Hu, Kang Yang, Fang Yang, Liya Chen, Zeng Yang, You Long, Chao Shen, Bo Yao, Chenghong Huang

**Affiliations:** 1 School of Chemistry and Chemical Engineering, Chongqing University of Science and Technology, Chongqing 401331, China2022441980@cqust.edu.cn (L.C.); 2007013@cqust.edu.cn (B.Y.); 2 School of Pharmacy and Nursing, Chongqing Vocational College of Light Industry, Chongqing 401329, China

**Keywords:** CMCS, vanillin isomers, crystallization

## Abstract

The medicinal value of vanillin and its isomers has not been well developed, so it is necessary to prepare crystals of vanillin and its isomers as well as to investigate their crystallization rules in detail using advanced crystallization techniques in polymer gel. Based on molecular simulation, the maximum number of hydrogen bonds between CMCS with Van, IsoVan and oVan were reached at molar ratios of 1:9 and 1:4 and 1:5, respectively. The gel hardness and apparent viscosity of CMCS/Van isomers were proportional to the mole ratio, while elongation at break and tensile strength decreased with an increase in molar concentration depending on the position of the side chain group of the Van isomer, exposure of the benzene ring, steric resistance and the number of hydrogen bonds formed. The crystallization of Van, IsoVan and oVan in CMCS gel unexceptionally follow classical supersaturation theory in the case that nVan mainly exhibits a unique growth pattern from needle to strip, IsoVan’s growth style changes from plate to bulk and oVan adapts growth pattern from needle to branch bifurcating. It was also found that the Van crystal changed from II-type to I-type under long-term heating. Studies have further confirmed that the discrepancy of physicochemical characteristics of CMCS/Van blend gel can be attributed to differences in the number of hydrogen bonds compared to CMCS with given group positions of Van isomers. This study provides powerful technical support for the gel crystallization of van isomers.

## 1. Introduction

**Figure 1:**
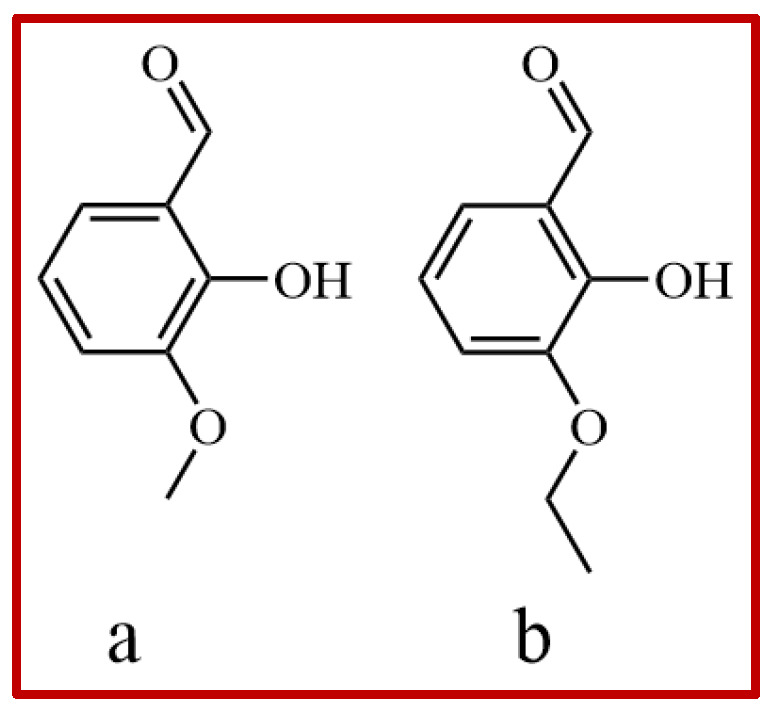
Chemical structure of vanillin ((**a**), van) and ethyl vanillin ((**b**), EVA).

**Figure 2:**
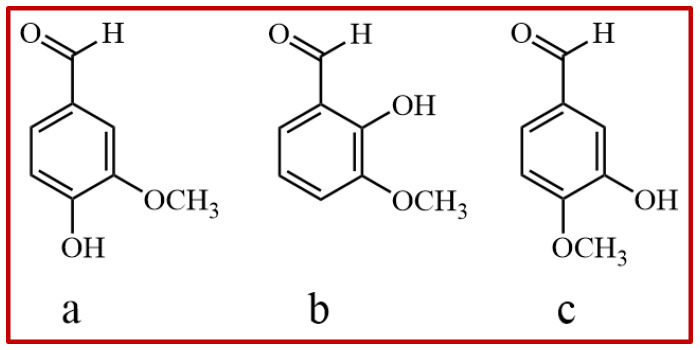
Chemical structure of n-vanillin ((**a**), Van), ortho-vanillin ((**b**), oVan) and iso-vanillin ((**c**), IsoVan).

Vanillin (Van), chemically named 4-hydroxy-3-methoxy benzaldehyde [1] with a molecular formula of C_8_H_8_O_3_, is ordinarily sourced from both natural and synthetic origins [2]. Van, together with ethyl vanillin (EVA) (Figure 1), is primary component of vanilla bean extract, and is usually used as a flavoring agent [3], antimicrobial agent [4], anti-inflammation agent [5], anti-cancer drug, [6] antioxidant agent [7] and so on. Due to the excellent pharmacological activity of vanillin [8], it has been widely welcomed by the industry to recover and purify as an active pharmaceutical ingredient (API) for the development of new preparations. Van has three isomers including vanillin (Van), iso-vanillin (IsoVan, 3-hydroxy-4-methoxybenzaldehyde) and ortho-vanillin (oVan, 2-hydroxy-3-methoxybenzaldehyde) [9,10] (Figure 2). Previous studies on their pharmacological value had mainly focused on Van [11,12,13,14]. Recent studies have further discovered that the other two isomers together with their derivatives are also of great economic value [15,16]. Therefore, it is necessary to develop a simple crystallization technology to obtain pure isomers for more pharmacological studies and formulation development.

Crystals, particularly in the context of pharmaceuticals, offer several advantages for drug formulation, delivery and performance, including an improved solubility, enhanced stability, controlled drug release and reduced side effects. Therefore, crystallization is an important method to prepare drug APIs [17,18]. Currently, a significant number of crystallization techniques such as the supersaturation rapid crystallization method (SRCM) [19,20] in a pure solution or mixed solution, liquid–liquid phase separation crystallization (LLPS) [21], template-mediated crystallization techniques (TMCT) [22], the vapor diffusion crystallization technique (VDRT) [23], polymer crystallization (PC) [24], etc., are utilized to produce Van crystals. However, they have several drawbacks including a long operation time, tedious operations and inherent flaws. Furthermore, the industrial preparation of Van and EVA is mainly obtained through oxidation [25], acid/alkali hydrolysis [26] or biorefinery separation [27], which produce a large amount of industrial wastewater and causes great harm to the environment. Therefore, it is better to prepare them using an environmentally friendly gel method. In recent years, the use of polymer gel crystallization has attracted more attention from researchers due to the nanoconfinement effects [28] of formed polymer gel that can reduce the crystallization time and obtain flawlessness crystals, and can even allow for the realization of the selective crystallization of polymorphs [29] as the gel is thought to act as an inert matrix and may even be easily fractured for crystal recovery because it is a reversible process if the formed gel structures are ion-switchable and can be destroyed by a weak acid [30].

In 2023, we reported the preparation method of EVA crystals using sol–gel conversion by carboxymethyl chitosan (CMCS) polymer as gel-forming tool [31]. CMCS is a chemically modified form of chitosan. The modification involves the introduction of carboxymethyl groups (-CH_2_-COOH) into the chitosan backbone, rendering it more soluble and chemically reactive. Therefore, CMCS can form hydrogen bonds with compounds containing hydroxyl groups [32]. Assuming that the substance can be prepared by crystallization, it can form a large hydrogen bond network in CMCS sol and then crystallize rapidly to become a gel. The hydrogen bond network can then be destroyed by dilute hydrochloric acid and the corresponding crystals can be obtained by simple filtration. On the other hand, the promising value of Van isomers, an important API [33], is far from being exploited. In addition, mechanism of crystallization processes, particularly the structure and relationship between the performance of CMCS and Van isomers, are still unclear. Therefore, this study aims to investigate the crystallization rules for Van isomers within CMCS gel in detail. We expect that this study can provide a technical methodology for large-scale preparation of Van isomers.

## 2. Results and Discussions

### 2.1. Hydrogen Bonds from Molecular Simulation

**Figure 3:**
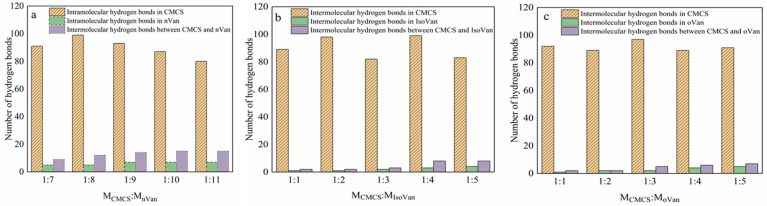
Formation of hydrogen bonds in CMCS/Van (**a**), CMCS/IsoVan (**b**) and CMCS/oVan (**c**) in the molecular simulation.

CMCS, prepared from chitosan through the introduction of a carboxymethyl group leading to the destruction of its secondary structure [34], almost behaved amorphously with the original crystal [32]. The CMCS molecule itself can form intra- and inter-molecular hydrogen bonds [34], and the hydroxyl group of CMCS can also form hydrogen bonds with the aldehyde group of vanillin isomers (Figure 3) [35]. Using the following conditions and parameter settings in the kinetic calculations for the CMCS and Van isomers, where MCMCS:MnVan = 1:7–1:11, MCMCS:MIsoVan = 1:1–1:5 and MCMCS:MOVan = 1:1–1:5, it was found that the number of both intra-molecule and inter-molecule hydrogen bonds were proportionally increased according to the mole ratio, which increased to a maximum of 1:9, 1:4 and 1:5, respectively. The local nanoconfinement formed by the large number of intra-molecular and inter-molecular hydrogen networks laid the foundations for the theoretical principle of the gel crystallization of Van isomers [36].

### 2.2. Transformation of Physical Chemical Performances During Sol–Gel Conversion

**Figure 4:**
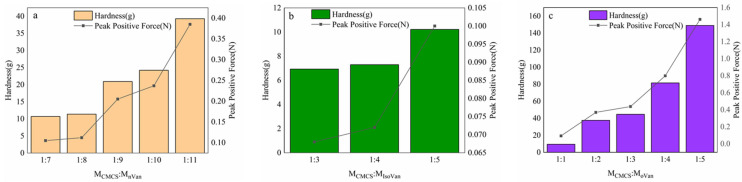
Test of gel hardness of blend gels of CMCS/Van (**a**), CMCS/oVan (**b**) and CMCS/IsoVan (**c**) with different mole ratios.

The apparent viscosities of the prepared 0.01% (*w*/*v*) CMCS sol measured at 100 rpm, 300 rpm, and 600 rpm were 9/mPa-s, 5/mPa-s, and 5.5/mPa-s, respectively. The pH value experienced no significant change. However, the rheological properties of the CMCS gels prepared using corresponding concentrations showed a better ductility and tensile strength, exhibiting a 0.8045% elongation at break and a 20.126 MPa tensile strength under the condition of an angular frequency of 9.5 rad/s with G″ > G′ (Figure 4). The gel hardness of the CMCS/Van isomer blends was enhanced with the increase in MVan and its peak positive force was synchronously correlated with the molar amount of Van added. CMCS polymer chains are maintained by hydrogen bond networks to form a polymer network structure [10]. With the addition of Van, the CMCS becomes more easily stacked with Van molecules due to increased Van molecule accumulation, giving rise to more H-bond cross-linking sites and hydrogen bonds with a more densely enclosure and an improved gel hardness performance [37]. Thereafter, water molecules can practically not penetrate into the interior of the three-dimensional structure. With the gradual formation of a hydrogen bond network and gel, the compartment of CMCS theoretically encloses the Van, creating a more ordered structure. Obviously, this works in favor of the rapid crystallization of Van in the gel. Otherwise, the hydroxy group in the IsoVan performs more actively as a result of the ectopic hydroxyl group and aldehyde group, which allow them to more easily form hydrogen bonds [38]. In addition to the hydrophobicity of the benzene ring, the IsoVans are inclined to carry out hydrophobic self-aggregation, which is very helpful for the rapid crystallization of Van in the gel at a mole ratio of 1:3–1:5. The rule for changes in gel hardness for CMCS/oVan blends is relatively similar to that for CMCS/Van in that a stronger gel corresponds to a higher oVan molecule number. The distinct difference is that oVan will slowly crystallize in gel as the self-aggregation of hydrophobic benzene ring has as stronger impact than that of carboxylate oxygen between CMCS and oVan [39].

**Figure 5:**
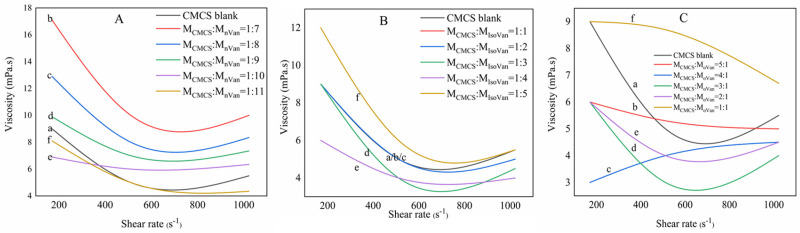
Viscosity curves of CMCS/Van ((**A**), mol ratio of of CMCS blank (a), 1:7 (b), 1:8 (c), 1:9 (d), 1:10 (e) and 1:11 (f)); CMCS/IsoVan ((**B**), mol ratio of CMCS blank (a), 1:1 (b), 1:2 (c), 1:3 (d), 1:4 (e) and 1:5 (f)) and CMCS/oVan ((**C**), mol ratio of CMCS blank (a), 1:1 (b), 1:2 (c), 1:3 (d), 1:4 (e) and 1:5 (f)).

Due to the existence of group-CH_2_COOH, CMCS sol itself shows high hydrophilic and thickening performances. The addition of Van into CMCS sol can encourage its hydroxyl to crosslink with hydrogen atoms in CMCS, leading to the generation of a large number of electrostatic attractions [39]. The CMCS/Van sol presents a typical shear-thinning pseudoplastic fluid feature meaning that its apparent viscosity (Figure 5) will descend with a decrease in the rate of shearing to lower than 650 s^−1^ for CMCS/Van, 690 s^−1^ for CMCS/Isovan and 620 s^−1^ for CMCS/oVan. Comparatively speaking, the hydrogen bond in CMCS/Van gel will break under the shearing force action accompanied by a reduced viscosity. Accordingly, the hydrogen bond can also be recombined by an increased viscosity. For CMCS/IsoVan gel, the viscosity will reduce at mole ratio of 1:1–1:4 but will reach up to maximum at 1:5. However, the situation is somewhat different for CMCS/oVan in that an equal number of hydrogen bonds will be obtained for a mole ratio of 2 but the viscosity will weaken with a rise in the rate of shearing to a mole ratio of 5.

**Figure 6:**
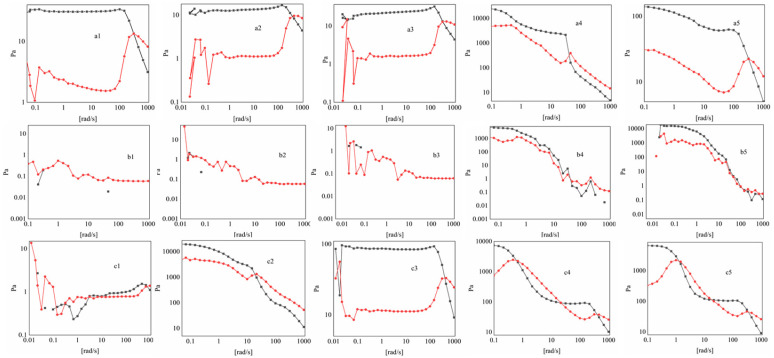
Energy dissipation and energy storage modulus of CMCS/Van ((upper section), mol ratio = 1:7 (**a1**), 1:8 (**a2**), 1:9 (**a3**), 1:10 (**a4**) and 1:11 (**a5**)); CMCS/IsoVan ((middle section), mol ratio = 1:1 (**b1**), 1:2 (**b2**), 1:3 (**b3**) 1:4 (**b4**), 1:5 (**b5**)) and CMCS/oVan ((lower section), mol ratio = 1:1 (**c1**), 1:2 (**c2**), 1:3 (**c3**) 1:4 (**c4**), 1:5 (**c5**)).

The measured elastic modulus of the CMCS/Van system (Figure 6) shows that elastic solid behavior predominates (G′ ≧ G″) at a fixed angular frequency of 10 rad/s. For specific critical strains, G′ may be equal to G″ or G′ ≦ G″. In contrast, the CMCS/IsoVan system exhibits a colloidal energy dissipation modulus of G′ ≧ G″ at molar ratios of 1:1–1:3, and G′ equals G″ at 1:4–1:5, and subsequently G″ become larger than G′. The CMCS/oVan system is highly similar to CMCS/Van.

**Figure 7:**
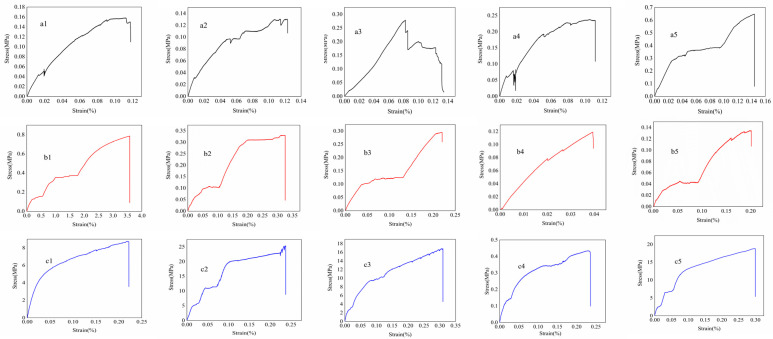
Stress–strain diagram of CMCS/Van isomers including CMCS/Van ((upper, mole ratio), 1:7 (**a1**), 1:8 (**a2**),1:9 (**a3**), 1:10 (**a4**) and 1:11 (**a5**)); CMCS/IsoVan (middle, mole ratio) together with oVan (lower, mole ratio) are 1:1 (**b1**,**c1**), 1:2 (**b2**,**c2**), 1:3 (**b3**,**c3**), 1:4 (**b4**,**c4**) and 1:5 (**b5**,**c5**).

The stress–strain relationship within the fabricated CMCS/Van film was further tested and characterized by a fractured growth rate and tensile strength (Figure 7). The results demonstrated that more strong hydrogen bonds or weak hydrogen bonds exist according to the different origins of the hydroxyl, carboxyl and aldehyde groups. The former confers resilience and the latter endows temporary cross-linking to the gel, which endue it a somewhat mechanical character [40]. The decrease in the elongation at break and tensile strength of the CMCS/Van film with the increase in molar concentration is mainly attributed to the Van crystal’s occupation of the hydroxyl group of CMCS. The difference is that the hydroxyl and aldehyde groups in Van, IsoVan and oVan have diverse positions, steric hindrances and hydrophobic forces resulting in the formation of hydrogen bonds which causes differences in gel hardness, thus affecting their tractility and mechanical strength. The worse tensile strength and break growth rate of the CMCS/oVan film can be ascribed to its more exposed benzene ring making it more hydrophobic, such that it performed uniform in the disperse tests, even in an oil precipitation in sol after long time placement.

### 2.3. Crystallization of Vanillin Isomers in CMCS Gels

**Figure 8:**
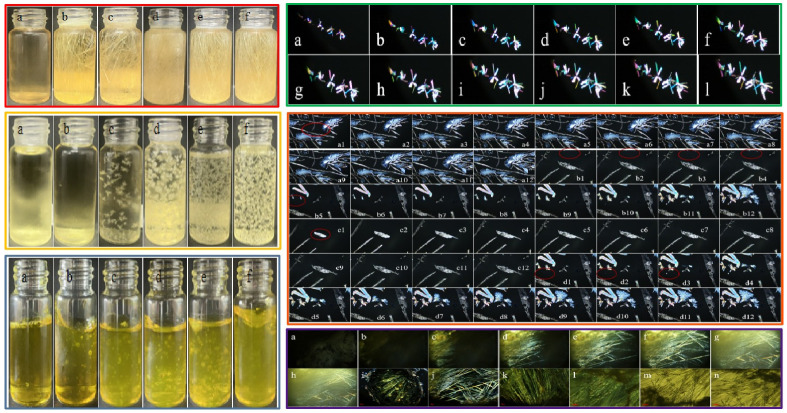
Crystallization process of Van in CMCS gel (red-line enclosed section) with mole ratio of (**a**) blank control, (**b**) 1:7, (**c**) 1:8, (**d**) 1:9, (**e**) 1:10 and (**f**) 1:11. Likeness to IsoVan (yellow-line enclosed section) and oVan (cyan-line enclosed section) in CMCS gel with an identical mole ratio of (**a**) blank control, (**b**) 1:1, (**c**) 1:2, (**d**) 1:3, (**e**) 1:4 and (**f**) 1:5. Above process were all performed at 4 °C. The green-line enclosed section (**a**–**l**), orange-line enclosed section and purple-line enclosed section (**a**–**n**) show the results of polarizing microscope observations of Van, IsoVan and oVan crystallization in CMCS gel showing different states of behavior. Detailed description can be seen from text.

Van, IsoVan and oVan, with corresponding thermodynamic ionization rates of 4.02 × 10^−8^, 1.29 × 10^−9^ and 1.23 × 10^−8^, can be dissolved in weak acid at 25 °C [41]. The crystallization process of Van isomers in CMCS gel is illustrated in Figure 8. At 4 °C, low concentration Van can rapidly precipitate crystals as shows in Figure 8 (green-line enclosed section) that a small number of needle-like crystals had already nucleated and precipitated at the edges up on observation through polarizing microscope, and crystals growth nearly ceased after 2 min. Van will not crystallize in CMCS gel within 168 h if the mole ratio of the CMCS/Van is lower than 7. Only millet-like crystal particles will be formed at the liquid–vapor interface at a mole ratio of 1:8. They then turn into needle-like crystals after 24 h growth. Crystals may appear within 6 h at a mole ratio of 1:9 and can even crystallize within 30 min at mole ratio higher than 1:10. Nevertheless, IsoVan can develop into a crystal after 12 h at mole ratio of 1:2 and it can transform into plate crystals at a higher mole ratio. At the gas-liquid interface on the edge of the CMCS gel, IsoVan is more likely to reach a supersaturated concentration, making its nucleation and crystallization process observable earlier. It can be seen that the crystal growth extends outward in all directions. Finally, it grows into a larger feathery crystal (a1–a12 in orange-line enclosed section) or branching out into gels (b1–b12 in orange-line enclosed section), either growth begins with nucleation at the center (c1–c12 in orange-line enclosed section) or presenting small granular crystals (d1–d12 in orange-line enclosed section).Inversely, oVan cannot crystallize from CMCS/oVan blend gel when the mole ratio is lower than 1:3 for less than 7 days. Crystals can uniquely appear at mole ratio higher than 1:4 after 25 h. Observations with a polarizing lens show that spherical, in small amounts linear, and linear crystals are slowly precipitated on small granular crystals for oVan in CMCS gels. The above results showed that the crystallization of Van, Isovan and oVan in CMCS gel is affected by molar ratio, temperature and time, and the crystallization behavior is quite different due to formation of different numbers of hydrogen bonds.

**Figure 9:**
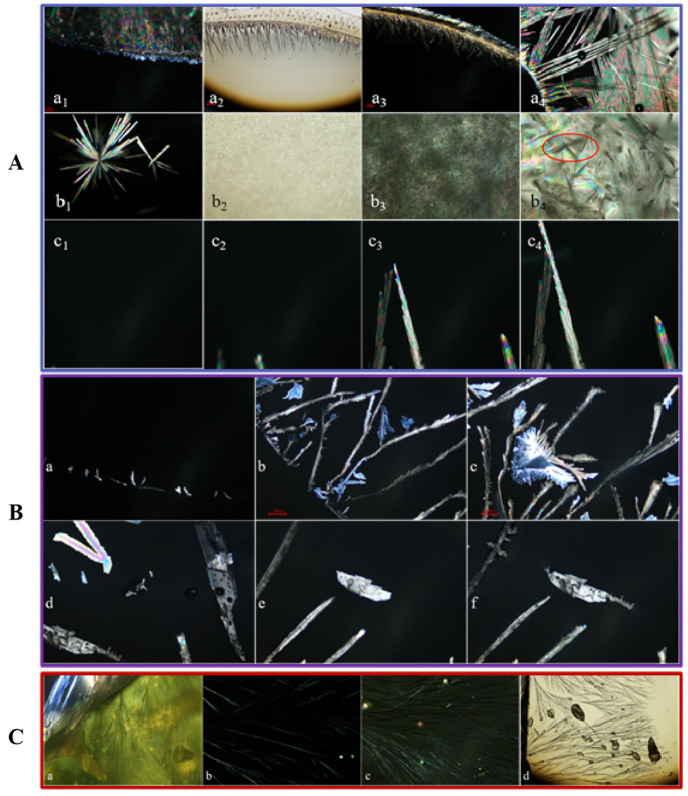
Polarizing microscope observations of Van crystallization behavior at gel edges (**a_1_**–**a_4_**) and center (**b_1_**–**b_4_**,**c_1_**–**c_4_**) in (**A**) section; IsoVan crystallization behavior observed by polarizing microscope at the edge (**a**–**c**) and center (**d**–**f**) of the gel in (**B**) section. Crystallization behavior of oVan at the edge (**a**) and center (**b**–**d**) of the gel observed by polarizing microscopy in (**C**) section.

The growth process of vanillin isomers crystal during CMCS’s transition from sol–gel at the micro-nano scale was observed at length by polarized microscopy (Figure 9). It was found that fine Van crystals with an arborization morphology will grow at the gas–liquid interface and randomly develop into bigger needle crystals along the edge within the gel’s internal gap. Typical and colorful polarizing effects may be observed at the bottom of the vessel. IsoVan can slowly form sheet crystals at the gas–liquid interface. Subsequently, it grows into the central region and slowly converts into a twisted configuration and finally develops into feathery granules. In some case it will expand into a plate configuration, on which a few little crystals will regrow without restrictions.

### 2.4. Crystal Recovery and Characterization

**Figure 10:**
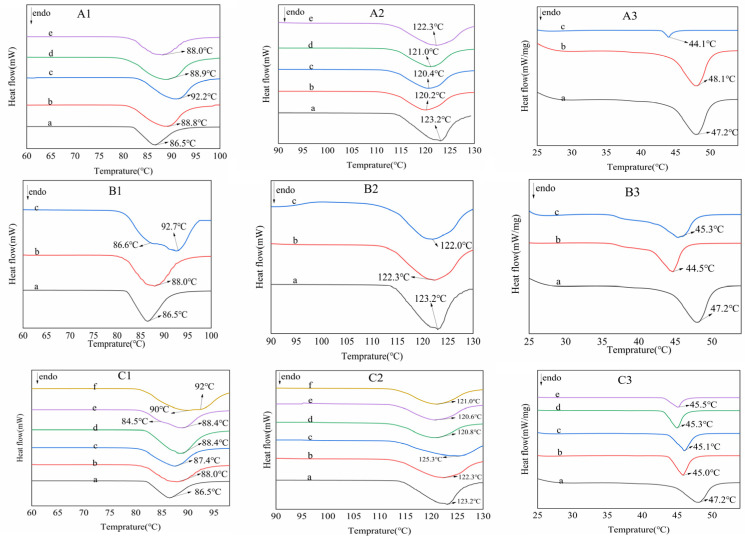
DSC spectra of different factors’ effects on Van crystals from CMCS gel ((**A1**), MCMCS:MVan = 1:1 (a), 1:2 (b), 1:3 (c), 1:4 (d) and 1:5 (e)); crystallizing temperature of ((**B1**) raw crystals (a) from CMCS gel at 4 °C (b) and 25 °C (c)), heating time ((**C1**) 0 min (a), 15 min (b), 30 min (c), 45 min (d), 60 min (e) and 120 min (f)). Likeness to IsoVan including MCMCS:MVan ((**A2**), 1:1 (a), 1:2 (b), 1:3 (c), 1:4 (d) and 1:5 (e)), crystallizing temperature ((**B2**) raw crystals (a) from CMCS gel at 4 °C (b) and 25 °C (c)), heating time ((**C2**) 0 min (a), 15 min (b), 30 min (c), 45 min (d), 60 min (e) and 120 min (f)) as well as that of oVan about mole ratio of ((**A3**), 1:1 (a), 1:2 (b), 1:3 (c)), crystallizing temperature of ((**B3**) raw crystals (a) from CMCS gel at 4 °C (b) and 25 °C (c)) and heating times ((**C3**) 0 min (a),15 min (b), 30 min (c), 45 min (d) and 60 min (e)), respectively.

The melting points of Van, IsoVan and oVan from purified commercially available materials following repeated crystallization are 80.5°, 116° and 43.5° [41]. Differential Scanning Calorimetry (DSC) results of recovered crystal samples are shown in Figure 10. There was only one endothermic peak produced for varied molar ratio of CMCS/Van, indicating that there is no polycrystalline phase change when heating to melting point. For crystallization with distinct temperature, it will give different results. At 25 °C (B1-c), Van DSC curve has two endothermic absorption peaks. The first peak appears at 86.6 °C, belonging to the melting point of type II metastable crystal, after which the type II metastable crystal began to melt and transformed into a type I crystal, leading to a second peak at 92.7 °C. It can be inferred that an increase in temperature may destroy the local hydrogen bond structure and then induce the crystalline form transformation of regular vanillin during crystallization, which is basically in accordance with the results reported by Ouyang [42]. The influence of heating time on crystal preparation is similar to that of temperature. However, there is only one endothermic peak for both IsoVan (Figure 10(A2–C2)) and oVan (Figure 10(A3–C3)) indicating that they both did not undergo polycrystalline phase transition.

**Figure 11:**
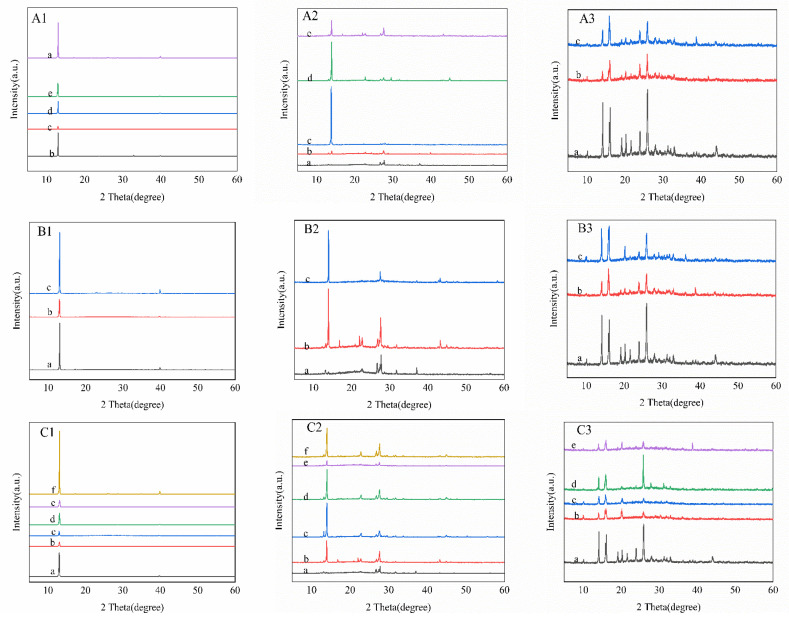
XRD spectra of Van crystals from different mole ratio of ((**A1**) MCMCS:MVan = 1:7 (a), 1:8 (b), 1:9 (c), 1:10 (d) and 1:11 (e)), temperatures with ((**B1**) raw material (a), crystal obtained from 4 °C (b) and from 25 °C (c)), heating times ((**C1**) for blank control (a), 5 min (b), 15 min (c), 30 min (d), 60 min (e), 120 min (f)). Likeness to identical conditions for Iso Van crystals that of (**A2**–**C2**) and for oVan that of (**A3**–**C3**).

The results from the X-ray diffraction (XRD) determination of Van are shown in Figure 11(A1–C1), revealing characteristic diffraction peaks at 2θ values of 13.303°, 17.76°, 23.707°, 26.997° and 39.9°, respectively. These results also explain that Van crystal from CMCS gel belong to II but can fall into I by heating at 25 °C for one hour. The IsoVan crystal presents typical diffraction peaks at 2θ values of 13°, 23°, 27°, and 28° (Figure 11(A2–C2)). The different feathery, plate and granule crystal of IsoVan possess identical crystal forms. It is presumed that the formation of the feathery Van crystal was due to the accumulation of small plate IsoVan that will spiral growth owing to nano-limit effect and hydrophobic self-aggregation of Van molecules and that formation of particle crystals was due to precipitation effect by supersaturation of rapid cooling as well as poor nucleation by rapid nucleation [40]. Diffraction characteristic peaks of oVan exhibited at 2θ angle of 10.171°, 14.182°, 16.161°, 22.49°, 26.048° that is basically consistent with the results in literatures [20,43].

## 3. Conclusions

This study systematically elucidated the nucleation and crystallization rule of vanillin isomers in CMCS gel and established a feasible technology roadmap of crystal preparation for van isomers. Based on molecular simulation, number of hydrogen bonds between CMCS with Van, isoVan and oVan were reached up to maximum at molar ratio of 1:9 and 1:4 and 1:5, respectively. Gel hardness and apparent viscosity of CMCS/Van isomers are proportionally to mole ratio, while elongation at break and tensile strength decreased with increasement of molar concentration depending on the position of the side chain group of the Van isomer, the exposure of benzene ring, steric resistance and the number of formed hydrogen bond. Van, isoVan and oVan entirely follow classical supersaturation theory for crystallization in that case Van mainly performed unique growth pattern from needle to strip, isoVan took growth style from plate to bulk and oVan adopted growth pattern from needle to branch bifurcating. It was also found that the Van crystal changed from II-type to I-type under long heating. Studies further confirmed that the discrepancy of physicochemical characteristics of CMCS/Van blend gel can be attributed to differences of number of hydrogen bonds from CMCS with specific group positions of Van isomers. This study can provide powerful technical support for van gel crystallization.

## 4. Experimental

### 4.1. Instruments and Reagents

#### 4.1.1. Instruments

The instruments used were offered by the following: Rotational Viscometer (ZNN-D-6), Qingdao Senxin Electromechanical Equipment Co., Ltd., Chongqing, China; Electronic Universal Testing Machine (CMT-1202), Zhuhai Sansi Tage Electrical Equipment Co., Ltd., Chongqing, China; Antonpa Rheometer (MCR102), Antonpa (Shanghai) Trading Co., Ltd., Chongqing, China; Texture Analyser (TA. XT Plus990000), Shanghai Ruifen Intelligent Technology Co., Ltd., Chongqing, China; FT-IR (Nicolet iS 10), Agilent Technology (China) Co., Ltd., Chongqing, China; XRD diffractometer (XRD-7000S/L), Beijing East–West Analytical Instruments Co., Ltd., Chongqing, China; DSC calorimeter (DSC35000A-1591-L), German Nitro Instruments Manufacturing Co., Ltd., Chongqing, China; Polarising microscope (BX53), Olympus Corporation, Tokyo, Japan.

#### 4.1.2. Reagents

Chitosan and Carboxymethyl chitosan (CMCS, MW/150,000) were both purchased from Shanghai Yuanye Biotechnology Co., Ltd., Shanghai, China. Van, IsoVan and oVan were all bought from Shanghai Aladdin Biotechnology Co., Ltd., Shanghai, China.

### 4.2. Molecular Simulation

The molecular formulas of CMCS and Van isomers were firstly drawn using the Materials Studio (MS) software 2020, in which the degree of polymerization (DP) of CMCS was set to 10 for computational feasibility during calculation. Initial structure optimization was performed using the DMoL3 module with energy minimization to achieve the lowest-energy conformation. A simulation box was constructed using the amorphous cell module, containing water molecules, CMCS and vanillin isomers in predefined stoichiometric ratios. Further geometry optimization was conducted using the Forcite module with the COMPASS force field to refine atomic interactions. The system underwent 100 annealing cycles (300–500 k) to enhance conformational sampling and eliminate high-energy configurations, where the parameters were set as a temperature of 298 K using the NVT system and a 1.0 fs step size.

### 4.3. Preparation of CMCS Blank Gels and CMCS/Van Isomer Blend Gels

The preparation of CMCS blank gels and CMCS/Van isomer blend gels was carried out according to the following method reported in the literature [31]: weigh CMCS (0.01%, *w*/*v*) into 50 mL deionized water, then heat to 80 °C with 600 rpm magnetic stirring until the CMCS completely dissolves. The blends were dispensed into vessels and cooled to room temperature, before finally being placed in a refrigerator at 4 °C to cool till to gel formation. Triplicate parallel experiments were performed.

### 4.4. Determination of Sol Viscosity

The sol was heated at 80 °C in a six-speed apparatus, and the viscosity was measured at 100 rpm, 300 rpm and 600 rpm, respectively, and then readings were taken to calculate the apparent viscosity at the corresponding speeds using Equation (1).

(1) $$\mu_{a}=\frac{\tau }{\gamma }=\frac{0.511^{\theta }N}{1.703N}\times 1000=\frac{300^{\theta }N}{N}$$

In the formula, *Ν* denotes rotational speed, r/min; ᶱ*N* denotes the dial reading when the rotational speed is *N*; and *μ_a_* is the apparent viscosity, mPa·s.

### 4.5. Measurement of Rheological Properties

The storage modulus and dissipation modulus were measured by a rheometer with an angular frequency of 10 rad/s and an amplitude of 0.01~1000.

### 4.6. Measurement of Tensile Strength

The sol was placed in an ultra-clean bench at 25 °C until it was completely dried to form a film, then removed and cut into a 1 × 4 cm film, and after measuring the thickness and initial length with vernier scale, it was subjected to tensile test at a speed of 0.1 mm/min. The tensile force and elongation of the gel film at rupture were recorded. The elongation at break was calculated as follows:

E = (L − l)/l × 100%(2)

In the formula, E—elongation at break, %; L—distance between marks when sample breaking, mm; l—sample original mark distance, mm.

Tensile strength was calculated as follows:

TS = F/(a × b)(3)

TS—tensile strength, MPA;
Maximum tensile force, N;
a—width, mm;
b—thickness, mm.

### 4.7. Gel Hardness Test

The sol was placed in a gel mold with an inner diameter of 20 mm × 23 mm, refrigerated at 4 °C until it became a gel and transferred to a texture analyzer; we then selected the TPA compression mode with a probe of P/0.5, a post-test speed of 4 mm/s, a compression distance of 10 mm, a compression time interval of 5 s between the two compression times and an induced force of 5 g.

### 4.8. DSC Test

A 5.0 mg sample was taken and sealed in a crucible. The sample’s heat spectrum was measured and recorded using a differential scanning calorimeter (DSC) at 10 °C/min in nitrogen with a flow rate of 25 mL/min.

## Data Availability

The original contributions presented in this study are included in the article. Further inquiries can be directed to the corresponding author.
